# Case Report: Mucoepidermoid carcinoma of the lung, a challenging diagnosis of an unusual case

**DOI:** 10.3389/fonc.2025.1626604

**Published:** 2026-01-06

**Authors:** María C. Garijo Martínez, Alejandro Olivares-Hernández, Lorena Bellido Hernández, Fernando L. Begliardo, Daniel Morchón-Araujo, Jonnathan Roldán Ruiz, Juan Carlos Redondo González, Luis Posado Domínguez, Eva Campaña Díaz, Inés María de Dios Franco, Emilio Fonseca Sánchez, Edel del Barco Morillo

**Affiliations:** 1 Department of Medical Oncology, University Hospital of Salamanca, Salamanca, Spain; 2 Biomedical Research Institute of Salamanca (FIBSAL), Salamanca, Spain; 3 Faculty of Medicine, University of Salamanca (USAL), Salamanca, Spain; 4 Department of Radiology, University Hospital of Salamanca, Salamanca, Spain; 5 Department of Nuclear Medicine, University Hospital of Salamanca, Salamanca, Spain; 6 Department of Pathology, University Hospital of Salamanca, Salamanca, Spain

**Keywords:** lung cancer, mucoepidermoid carcinoma, salivary glands, rare neoplasm, low-grade, case report

## Abstract

**Background:**

Lung cancer comprises several types of tumors, with the most common subtypes being adenocarcinoma and squamous cell carcinoma. Mucoepidermoid carcinoma of the lung is an extremely rare entity, whose diagnosis and treatment are challenging.

**Case presentation:**

We reported a 55-year-old woman who was diagnosed in the context of a respiratory disease with a mucoepidermoid carcinoma of the lung. The diagnosis was not easy due to the difficulty in obtaining a correct tissue sample to study. After discussing the case in a multidisciplinary tumor board, the patient underwent surgery with the exceptional diagnosis of a low-grade mucoepidermoid carcinoma of the lung. Since then, the patient has been on follow-up, with no evidence of disease.

**Conclusions:**

There are some subtypes of lung cancer with very different behavior from the classical ones. For example, mucoepidermoid carcinomas of the lung have an exceptional survival with surgery, without needing further treatment, when they are classified as low-grade. This case report underscores the importance of an appropriate diagnosis based on the work of multidisciplinary tumor boards, even when there are difficulties in obtaining a tissue sample, to offer to the patients the best opportunities.

## 1 Introduction

Mucoepidermoid carcinoma has traditionally been classified as a malignant tumor of the salivary gland. However, there have also been documented few cases of mucoepidermoid carcinomas manifesting in the lung. These neoplasms are exceedingly rare, accounting for 0.1-0.2% of all lung cancers. A distinctive feature of mucoepidermoid carcinoma is its predilection for younger patients, a phenomenon that contrasts with the expected characteristics of patients affected by this type of neoplasm, such as adenocarcinoma or squamous cell carcinoma. There is no clear risk factors identified, and the significance of established risk factors such as tobacco use is not completely known. Two distinct groups of this entity can be distinguished: high-grade and low-grade carcinomas. The latter are associated with an excellent prognosis, and surgery is the cornerstone of the treatment. Here we present a clinical case of a 55-year-old female patient diagnosed with low-grade mucoepidermoid carcinoma of the lung.

## 2 Case description

A 55-year-old Spanish woman at the time of diagnosis. She was diagnosed with stage IVa Non-Hodgkin Lymphoma in 2001. She received treatment with chemotherapy (scheme of CMF and FC), which resulted in a complete response. Initially, interferon was administered as a maintenance therapy, but it had to be discontinued due to toxicity. The patient had a history of tobacco use, with daily consumption of 30 cigarettes per day, and a 60 pack-year index. Her family history included: her father was diagnosed with esophageal cancer at the age of 58, her mother with colorectal cancer at the age of 65 and her sister with papillary thyroid cancer at the age of 25.

In November 2022 she was diagnosed with a viral respiratory infection. She had no improvement with the initial therapy with non-steroidal anti-inflammatory drugs and bronchodilators, so she went to the Emergency Department. She expressed persistent cough and fever above 39°C for two days. On the physical examination, the patient had a good performance status, with hypophonesis in left lung, with the remaining physical examination being normal. Her temperature was 37.2°C, with a heart rate of 97 beats/min and 20 breaths/min. Her blood pressure was 86/49 mmHg, and her oxygen saturation was 91% on room air. Laboratory results showed elevated acute-phase reactants as follows: elevated white blood cell count of 16.07 x 10^3^/µL (normal value 1.8–7 x 10^3^/µL), an increase percentage of neutrophil of 85.4% and a significant increased C-reactive protein level of 25.38 mg/dl (normal value ≤ 0.5 mg/dl).

**Figure 1:**
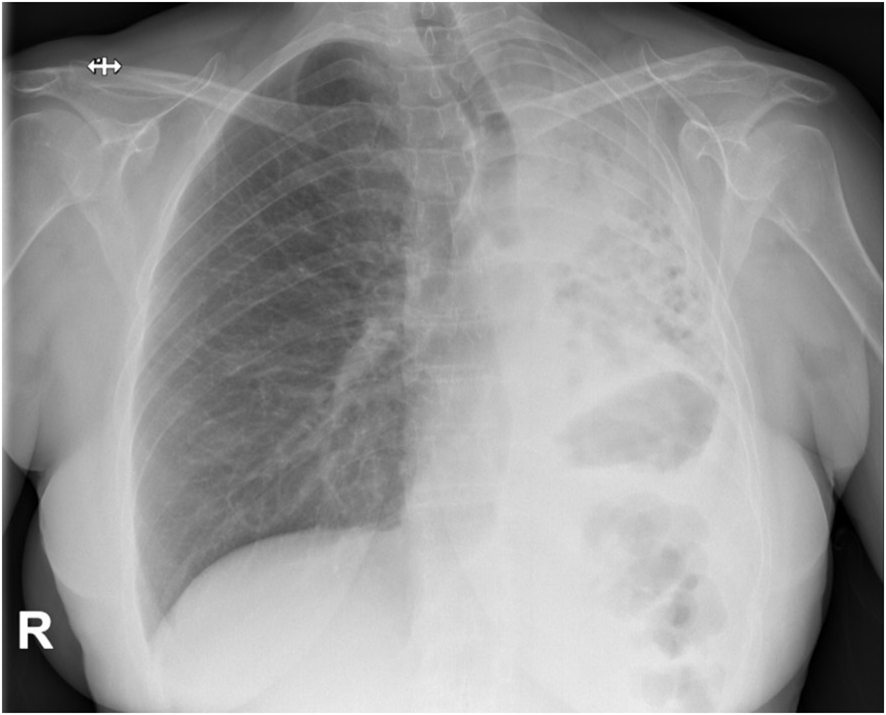
Chest X-ray of the patient at the emergency department with complete atelectasis of left lung.

**Figure 2:**
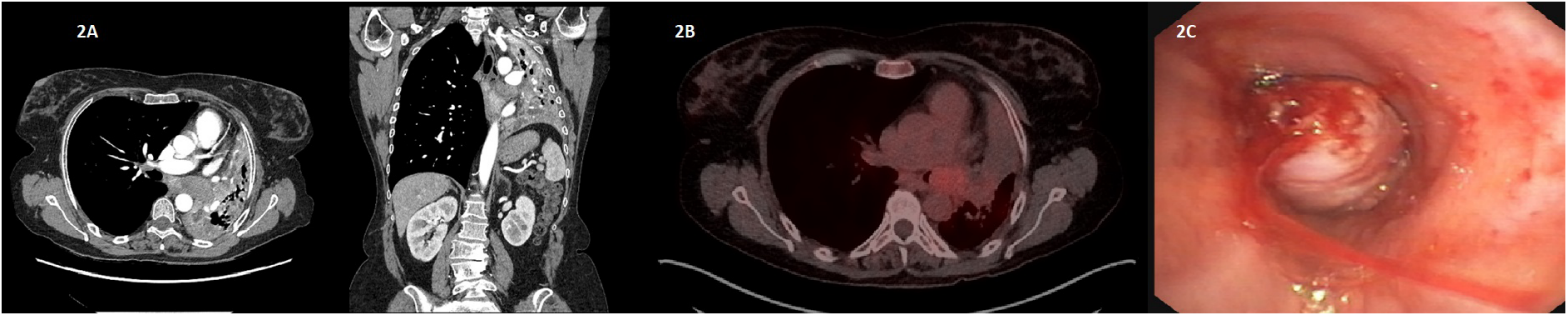
**(A)** Computarized tomography (CT) of the chest showing the endobronchial mass with associated atelectasis. **(B)** Axial representation of the FDG PET/CT of the patient showing the lung lesion with low tracer uptake. **(C)** Capture from the bronchoscopy test with the endobronchial lesion.

A chest radiography revealed complete atelectasis of the left lung (**Figure 1**). The patient was admitted in order to continue the diagnosis. During hospitalization, the patient had excellent performance status, without complications and a total recovery from the viral infection. A chest-abdomen-pelvis contrast-enhanced computed tomography (CT) was performed, showing an endobronchial lesion in the main and lower left lobar bronchi, measuring 3.8x2.2x3.2 cm, with associated atelectasis and pathological subcarinal adenopathies (**Figure 2A**). The lesion was located 1.5 cm from the carina and had moderate contrast enhancement.

To obtain further information, a PET/CT was performed, which described the lung lesion with low metabolic activity (SUVmax of 3.9), causing total obstruction of main left bronchi and subcarinal adenopathies with mild activity, without evidence of distant metastasis. It also was described some bronchiectasis with some areas associated with infection/inflammation (**Figure 2B**). Bronchoscopy revealed a whitish, irregular lesion at the bifurcation of the main bronchus towards the upper and lower lobar bronchi. Despite bronchoscopy being performed in two separate times, adequate histological analysis could not be obtained from the samples (**Figure 2C**).

**Figure 3:**
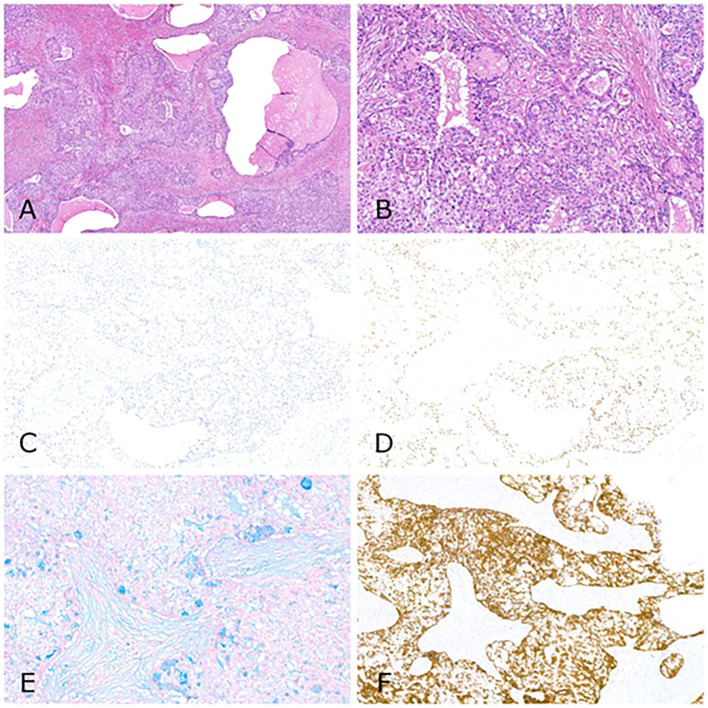
Mucoepidermoid Carcinoma of the Lung (WHO 2021). **(A)** H-E stain, low magnification (4x): low-grade tumors consist of varying proportions of mucin-secreting, squamoid, and intermediate cells. Cystic areas are typically lined by mucin-secreting cells. Solid areas are composed of intermediate cells and/or non-keratinizing squamoid cells. **(B)** H-E stain, intermediate magnification (10x). **(C)** TTF-1 immunohistochemistry (10x). **(D)** p40 immunohistochemistry (10x). **(E)** Periodic acid–Schiff with Alcian Blue (PAS-CB) stain (10x). **(F)** Cytokeratin 5 (CK5) immunohistochemistry (10x).

After deliberation by the multidisciplinary tumor board, the patient underwent a left pneumonectomy and lymphadenectomy via thoracotomy in March 2023. The pathological examination was consistent with low-grade mucoepidermoid carcinoma of the lung, measuring 4.7cm in diameter, with no affected margins and no lymph node or vascular-lymphatic involvement (pT2 pN0) (**Figure 3**). Immunohistochemistry (IHC) analysis results were: TTF1 -, CK7 +, CK 5+, p63 +. Molecular study using *Next Generation Sequencing (NGS)* revealed no actionable alterations.

**Figure 4:**
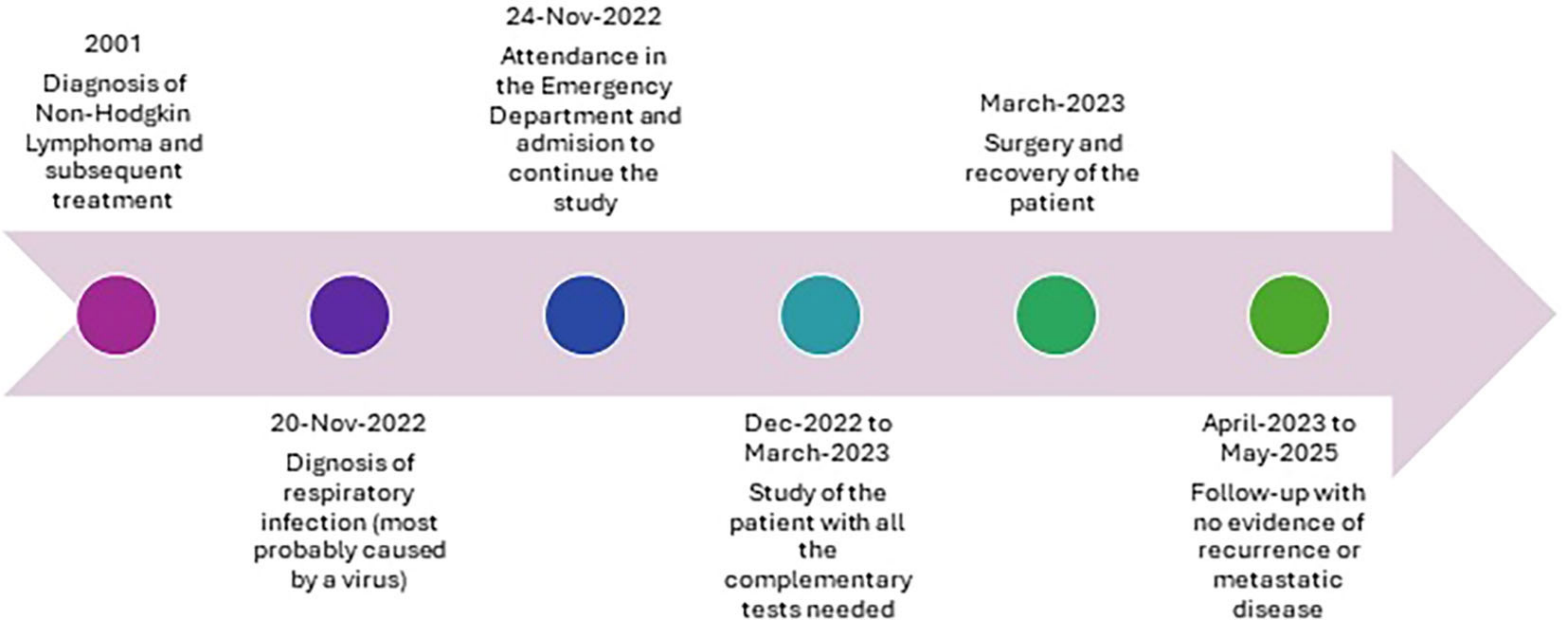
Timeline depicting main onco-hematologic events in our patient's life.

With this definitive diagnosis, the patient started monitoring, currently showing no evidence of disease (last follow-up in June 2025). Furthermore, she has an active lifestyle, with no aftermaths. In **Figure 4** there is a brief timeline with the main events in this case report.

### 2.1 Patient’s point of view

Once the diagnosis was established and the treatment performed, the patient recognized her anxiety and fear at the beginning of the study process. She was afraid of having one of the most common types of lung cancer, because of the short life expectancy associated with that diagnosis. However, she always trusted the team and the decisions taken by them in the multidisciplinary tumor board. She expressed gratitude for the multidisciplinary care she received on every visit to the hospital during her follow-up.

## 3 Discussion

Among primary lung tumors, there is a group like salivary glands neoplasms, which account for less than 1% of all lung cancer. The two main subtypes are adenoid cystic carcinoma and mucoepidermoid carcinoma comprising both of them more than 90% of all salivary glands neoplasm in the lung (1, 2). In a systematic review, it was stablished that Mucoepidermoid carcinoma is around 56.6% of all (3, 4).

Mucoepidermoid carcinoma is an extremely rare neoplasm, representing approximately 0.1-0.2% of all lung neoplasms (5, 6). Due to its low frequency, there is limited evidence available on this entity, with only a small number of clinical cases published. It was first described in 1952 by Smetana et al. (7). This tumor usually affects young individuals, with cases diagnosed between the ages of 3 and 78 years (8). There is no significant sex predilection, although there may be a slightly male predominance. In addition to these characteristics, this type of tumor differs from other common histologies of lung cancer because it is not associated with typical risk factors such as exposure to radon, asbestos or smoking (4).

Regarding its clinical presentation, mucoepidermoid carcinoma does not have specific symptoms. This kind of tumors usually appears in the central airways, so the patients can have symptoms of obstruction (3, 9). It commonly presents with fever, cough, purulent sputum, hemoptysis and chest pain. At first, it can be mistaken with a cold or pneumonia, but in cases of respiratory infections that do not respond well to standard treatment, the possibility of this tumor should be suspected (10–13).

The first diagnostic test performed is typically a chest X-ray, which can reveal consolidations, atelectasis and nodules. The study is then completed with a chest CT scan and a bronchoscopy with biopsy. Obtaining a good-quality sample can be difficult in some cases, but it is essential to have accurate histological information before considering therapeutic options, especially to reject others diagnosis. Although the diagnosis is often made at early stages, it is crucial to perform a proper staging to rule out distant metastasis (2, 5, 10). It is very important to highlight, the psychological effects of the diagnosis process in the patients. One of the main concerns of our patient during the diagnosis was the possibility of been diagnosed with a more aggressive tumor, and with a worse prognosis. Psychological help may be needed in these cases to manage all the different emotions they can feel.

The tumor originates in the submucosal glandular tissue of the proximal airways, specifically in the trachea and bronchi, and typically extends to the segmental bronchi (11). The neoplasm is comprised of a mixture of mucus-secreting cells, epidermoid cells, and intermediate cells. Its immunohistochemical profile is consistent with that of our patient, showing negative TTF1 and positivity for CK7, p40, and p63 (2, 14). These tumors are subclassified in high or low-grade, based on the presence or absence of nuclear pleomorphism, necrosis, and mitotic activity. Low-grade tumors, which are the most frequent subtype, have an excellent prognosis, with a 5-year survival rate of up to 95%, which significantly differs from that of high-grade tumors, whose survival rate is 25-31% (5, 7).

As previously mentioned, the vast majority of mucoepidermoid carcinomas of the lung are low-grade and are diagnosed at early stages. Surgery, with both conventional techniques and more advanced methods, is considered the cornerstone of treatment (15). Subsequent management depends on the histological subgroup. For low-grade tumors, adjuvant treatment is not necessary (7, 11). However, in the case of high-grade tumors, clear recommendations cannot be made regarding the use of adjuvant chemotherapy or radiotherapy due to the limited available evidence. Furthermore, in cases of recurrent, advanced, or metastatic disease, there are no clear recommendations on the treatment to administer. It has documented poor responses with conventional treatments, including chemotherapy and radiotherapy (3).

As in other tumors, the molecular biology is taken more and more importance in Mucoepidermoid Carcinoma of the lung. In some patients, alterations in the epidermal growth factor receptor (*EGFR*) gene have been observed, including both mutations and receptor overexpression, with variable responses to different *EGFR* inhibitors. They have been described in 10.4% to 40% of all lung cases of this subtype (1, 3). It is being explored the use of target therapy in these situations, favored by the durable partial response of a patient with Mucoepidermoid lung carcinoma with an alteration of EGFR treated with gefinitib (16). To improve the prognosis of these patients, especially of the high-grade tumors, molecular investigation is being performed, in order to develop targeted therapies. One example is the *t(11;19)MECT1-MAML2* (1, 2, 11).

In conclusion, mucoepidermoid carcinoma is a rare form of lung cancer. It is imperative to develop a high degree of diagnostic suspicion and to confirm the diagnosis through an appropriate biopsy. The tumor grade is a pivotal factor in determining patient prognosis and the subsequent therapy. High-grade cancers have a poor survival outcome, so, molecular characterization is essential to identify potential therapeutic targets.

## Data Availability

The raw data supporting the conclusions of this article will be made available by the authors, without undue reservation.
